# ZnCr_2_O_4_ Nanoparticles: Facile Synthesis, Characterization, and Photocatalytic Properties

**DOI:** 10.1038/srep20071

**Published:** 2016-02-01

**Authors:** Zahra Mousavi, Faezeh Soofivand, Mahdiyeh Esmaeili-Zare, Masoud Salavati-Niasari, Samira Bagheri

**Affiliations:** 1 Institute of Nano Science and Nano Technology, University of Kashan, Kashan, P. O. Box. 87317-51167, I. R. Iran; 2 Nanotechnology & Catalysis Research Centre (NANOCAT), IPS Building, University of Malaya, 50603 Kuala Lumpur, Malaysia

## Abstract

In this work, zinc chromite (ZnCr_2_O_4_) nanostructures have been synthesized through co-precipitation method. The effect of various parameters such as alkaline agent, pH value, and capping agent type was investigated on purity, particle size and morphology of samples. It was found that particle size and morphology of the products could be greatly influenced via these parameters. The synthesized products were characterized by field emission scanning electron microscopy (FESEM), X-ray diffraction (XRD), fourier transform infrared (FT-IR) spectra, X-ray energy dispersive spectroscopy (EDS), photoluminescence (PL) spectroscopy, diffuse reflectance spectroscopy (DRS) and vibrating sample magnetometry (VSM). The superhydrophilicity of the calcined oxides was investigated by wetting experiments and a sessile drop technique which carried out at room temperature in air to determine the surface and interfacial interactions. Furthermore, the photocatalytic activity of ZnCr_2_O_4_ nanoparticles was confirmed by degradation of anionic dyes such as Eosin-Y and phenol red under UV light irradiation. The obtained ZnCr_2_O_4_ nanoparticles exhibit a paramagnetic behavior although bulk ZnCr_2_O_4_ is antiferromagnetic, this change in magnetic property can be ascribed to finite size effects.

Spinel compounds have a general formula AB_2_O_4_, in which the A-site is tetrahedrally coordinated and generally occupied by divalent cations (Mg, Mn, Ni, and Zn) and the B-site is octahedrally coordinated and occupied by trivalent cations (Al, Cr, and Fe). In solid-state science, oxides with spinel structures are some of the most studied compounds due to their wide range of applications. Spinels such as ZnCr_2_O_4_ containing transition metal ions can act as the efficient catalysts in the number of heterogeneous chemical processes such as carbon monoxide (CO) oxidation^1^, catalytic combustion of hydrocarbons^2^, reduction of several organic molecules^3^, sensing properties^4^ and effective photocatalysts^5^,^6^,^7^,^8^. The nanoparticles ZnCr_2_O_4_ have been synthesized by various methods including mechanical activation^9^, high-temperature solid-state reaction^10^, microemulsion method^11^, solution method^12^, and spray pyrolysis^13^. The use of low temperature chemical methods of synthesis has been a promising direction in the improvement of the technology for producing compounds with spinel structure.

Several chromites have been synthesized in nanocrystal form so far. ZnCr_2_O_4_ crystallizes well when the sintering temperature is above 500 °C^14^, and the product formed at 350 °C is in amorphous phase. Pure MgCr_2_O_4_ has been reported to have formed when an appropriate mixture of pure oxides is pressed into bars and sintered for several hours in an electric furnace at 1400 °C^15^. Some chromites, such as MgCr_2_O_4_, CuCr_2_O_4_, NiCr_2_O_4_, ZnCr_2_O_4_, and CoCr_2_O_4_, have been prepared using co-precipitation method, by the process of re-crystallization from pyridine followed by ignition in the temperature range of 700–1200 °C^16^. Nanometer-sized particles having perovskite structure were reported to be formed when partial substitution by alkaline rare earth metals was carried out in lanthanum chromites by urea combustion method, and this was followed by calcination at 900 °C^17^.

In this paper, we describe a precipitation method to synthesis ZnCr_2_O_4_ nanostructures with using Zn(NO_3_)_2_. 6H_2_O and CrCl_3_. 6H_2_O as starting materials. The aim of this work is to synthesize ZnCr_2_O_4_ nanostructures via a co-precipitation method and to investigate the effect of various parameters on their morphology. The photocatalytic activity of ZnCr_2_O_4_ nanoparticles (sample 4) was evaluated by the degradation of anionic dyes such as Eosin-Y and phenol red as water pollutants. Also, the photocatalytic degradation of anionic dyes such as Eosin-Y and phenol red using spinel ZnCr_2_O_4_ under UV irradiation at pH = 2–3 has been also examined.

## Results and Discussion

To investigate the role of different parameters such as alkaline agent, pH value and capping agent type on the morphology, purity, and particle size of the products, the various tests were done. All of the preparation conditions of the synthesized samples are presented in Table 1.

Recently, the role of capping agents in the morphology and size of nanomaterials has been studied, widely^18^. Hence, the different types of capping agent, including of poly vinyl pyrrolidone (PVP), cetyl trimethylammonium bromide (CTAB), and sodium dodecylbenzenesulfonate (SDBS) as polymeric and inert, cationic and anionic capping agents were applied, respectively.

**Figure 1:**
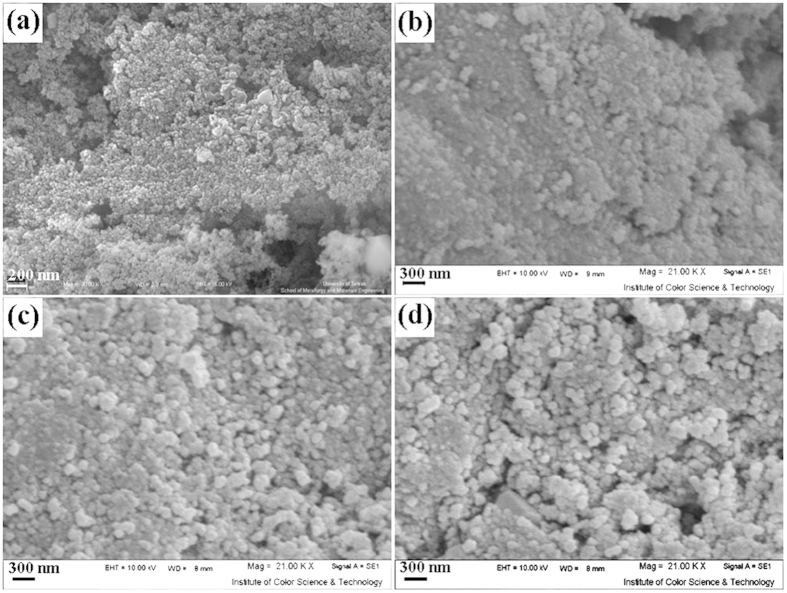
SEM images of sample (**a**) 1, (**b**) 2, (**c**) 3 and (d) 4.

Figure 1 shows the products synthesized in the absence and presence of various capping agents. In Fig. 1a, the 150 nm-sized agglomerated particles are shown that were related to blank sample (without capping agent), it is clear that aggregated nanostructures are formed in the absence of capping agent. According to the Fig. 1a, the particles coalesce and turn into bulk structures. Figure 1 (b–d) depicts the FESEM images of the sample 2, 3, and 4 synthesized by PVP, CTAB, and SDBS, respectively. As shown in Fig. 1b,d, the morphology of these samples are particles with average sizes about 70 nm. When PVP was used as capping agent, cubic structures are shown, additionally. Agglomerated and impacted particles obtained by using CTAB as a cationic capping agent. The formation of dense structures in Fig. 1c (sample 3) is due to its cationic head group, CTAB easily interact with free oxygen groups on the surface of nanoparticles and agglomeration of nanoparticles increases. So, SDBS as an anionic capping agent was chosen for achieving the desired product due to its homogeneity that is higher than the others.

**Figure 2:**
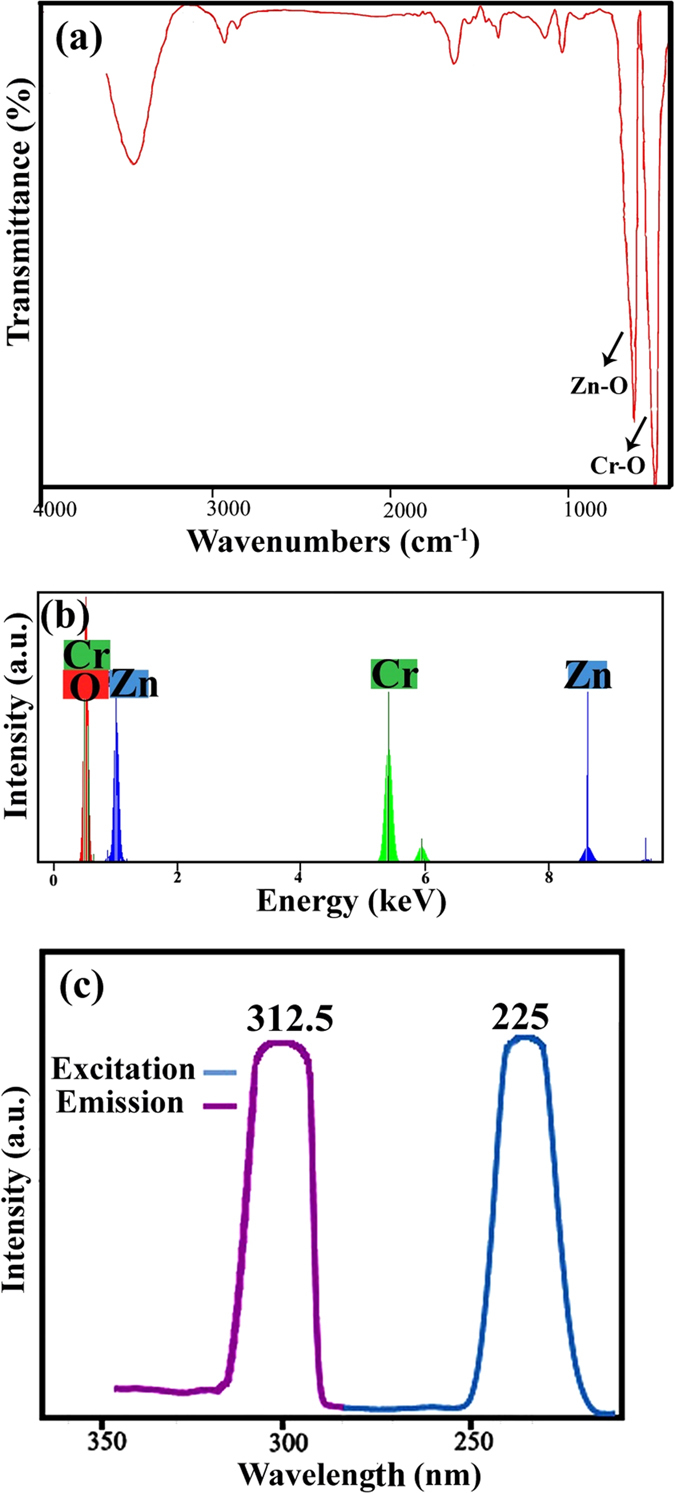
XRD patterns of sample (**a**) 1, (**b**) 2, (**c**) 3 and (**d**) 4.

The crystalline structures of the ZnCr_2_O_4_ nanoparticles were confirmed by XRD. Figure 2 (a–d) shows the XRD patterns of the sample 1–4, respectively. As shown in this figure, by utilizing of various capping agents can be achieved to ZnCr_2_O_4_ with different reference codes. All of peaks in these patterns (Fig. 2 (a–d)) can be indexed to cubic phase zinc chromium oxide with space group of Fd/3m (JCPDS: 73–1962, for sample 2 and 3) and (JCPDS: 22-1107 for sample 1 and 4). The crystallite sizes of the samples 1–4 were calculated by Scherrer equation^19^ and estimated about 78, 55, 28, 13 nm, respectively. According to this figure (Fig. 2d), in the presence of SDBS as capping agent (sample No. 4), the crystallinity of the obtained ZnCr_2_O_4_ is increased while in the absence of SDBS the crystallinity and purity are decreased.

ZnCr_2_O_4_ is a spinel with cubic phase, space group of Fd/3m and space group number 227. In this work, the all of the peaks in XRD patterns of samples can be indexed to cubic phase zinc chromium oxide but JCPDSs in these patterns are not the same. The various JCPDSs in the same phases of a compound are due to grain orientations of structures, for example: the principle ZnCr_2_O_4_ grain orientations are: (311), (200), (422), (440) for JCPDS =  = 22–1107; (311), (220) for JCPDS = 73–1962; and (220), (311), (511), (440) for JCPDS = 01–1123. Furthermore, the crystallographic parameters in various JCPDSs aren’t same, so in these patterns can be seen the different crystallographic parameters such as: a = 8.3257 in JCPDS = 22–1107; a = 8.28 in JCPDS = 73–1962; and a = 8.3200 in JCPDS = 01–1123 (cubic phase has a = b = c, and α = β = γ = 90°). By considering above explanations, the various JCPDSs in same phases of a compound can be used to determine grain orientations of structures.

**Figure 3:**
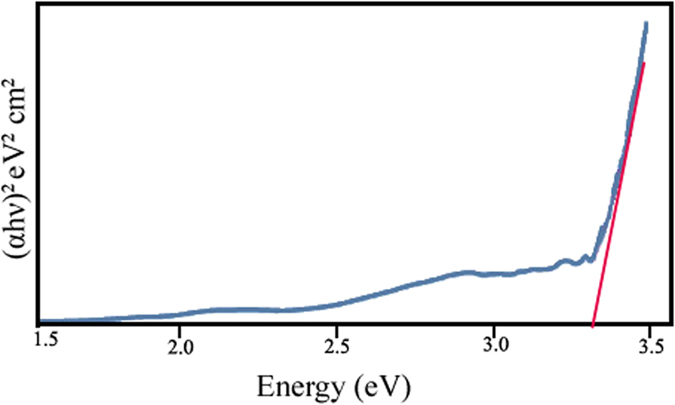
SEM images of sample (**a**) 4, (**b**) 5, (**c**) 6, and (**d**) 7.

To investigate the effect of alkaline agents on morphology of the products, SEM images of ZnCr_2_O_4_ were taken and shown in Fig. 3 (a–d). As shown in this figure, when sodium hydroxide (NaOH) was used as alkaline agent, the 70 nm-sized nanoparticles were obtained that smaller than the other alkaline agent (Fig. 3a). The size of particle obtained by using ammonia (NH_3_) (Fig. 3b), ethylene diamine (en) (Fig. 3c) and propylene diamine (pn) (Fig. 3d) are same and about 100 nm. When NaOH was used as an alkaline agent, pH of the reaction environment increases instantly and its reaction takes place with precursor rapidly, so, the nucleation process happens quicker than the growth step and fine particles are produced. The reactivity of NH_3_, en and pn is lower than NaOH, hence, the growth process was preferred to nucleation process and were produced larger particles.

**Figure 4:**
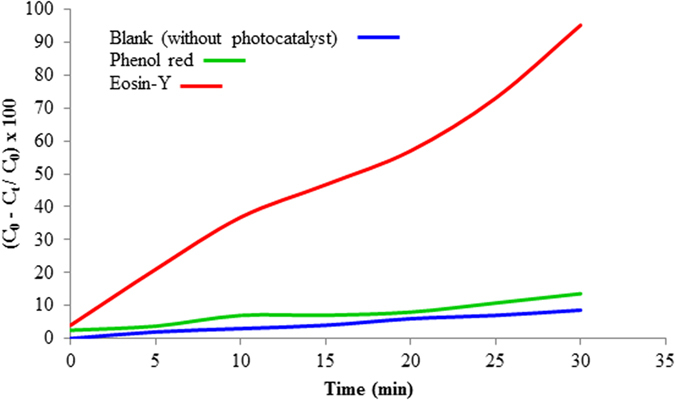
XRD patterns of sample (**a**) 5, (**b**) 6 and (**c**) 7.

Figure 4 (a–c) shows the XRD patterns of the samples synthesized in presence NH_3_, en, and pn, respectively. Figure 4a depicts the XRD pattern of sample 5, in this pattern is shown the mixture of ZnO (JCPDS card No. 80-0075), ZnCr_2_O_4_ (JCPDS card No. 01–1123; JCPDS card No. 73–1962) and unknown phases. By changing alkaline agent from NH_3_ (sample 5) to en (sample 6), all diffraction peaks (Fig. 4b) can be indexed to a pure cubic phase ZnCr_2_O_4_ (JCPDS card No. 22–1107) and ZnO was removed. Figure 4c is the pattern of the ZnCr_2_O_4_ in the presence of pn as an alkaline agent (sample 7). XRD pattern of sample 7 shows a pure cubic structure with space group and JCPDS card No. 73–1962, no characteristic peaks of other phases are observed. By comparing the XRD pattern of sample 4 to sample 5, 6, and 7 can be found that sample 4 is well crystallized and by considering their SEM images (Fig. 3) can be chosen NaOH as a desired alkaline agent for synthesis of pure ZnCr_2_O_4_ nanoparticles. The crystallite sizes of the samples 4, 5, 6, and 7 were calculated by Scherrer equation and estimated about 13, 33, 30, 27 nm, respectively.

**Figure 5:**
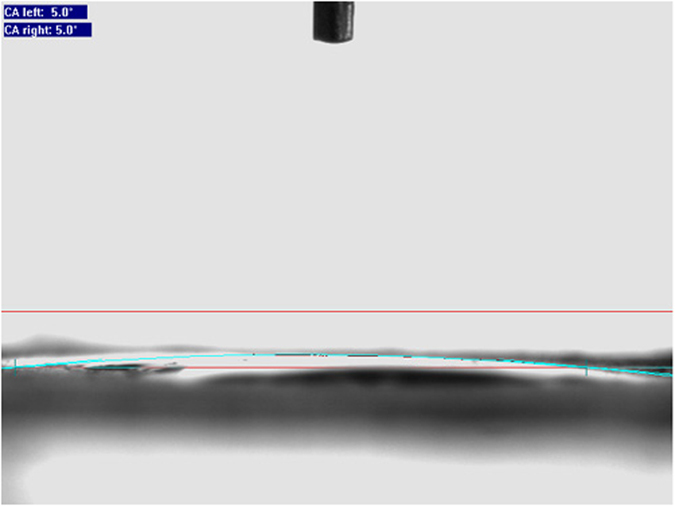
SEM images of sample (**a**) 4, (**b**) 8, and (**b**) 9.

The effect of the pH value on the morphology and purity of the products has been investigated. A series of experiments were done by decreasing the pH value from 14 to 11 and 7. Figure 5 (a–c) demonstrates the morphology of sample 4 (pH = 14), 8 (pH = 11), and 9 (pH = 7), respectively. By considering SEM images in Fig. 5 (a–c) can be observed that increasing pH led to the creation of smaller particles. It is obvious that the best nanoparticles are produced at pH = 14. Increase pH of solution can enhance nanoparticles surface charge and the electrostatic repulsive force. So, the agglomeration is decreased and the size of particles is being smaller. Surface charge and hydrodynamic diameter are two important properties of nanoparticle dispersions. The point where the surface charge density equals zero is defined as point of zero charge (PZC), while the point where zeta potential equals zero is defined as the isoelectric point (IEP)^20^,^21^. When a nanoparticle is dispersed in an aqueous solution, surface ionization and the adsorption of cations or anions result in the generation of the surface charge and an electric potential will be developed between the particle surface and the bulk of dispersion medium^22^,^23^.

The surface of nanoparticles dispersed in water is generally covered by the hydroxyl group as shown in Eq. 1. The surface charge of nanoparticles is a function of solution pH, which is affected by the reactions that occur on the particle surface as shown in Eqs. 2 and 3:


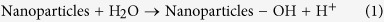



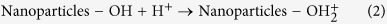



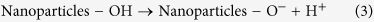


The pH at which the surface of nanoparticles is neutral is the point of zero charge or isoelectric point. If no specific adsorption of the ions presented in the solution takes place on the particle surface, the pH at PZC and IEP would be the same. When pH is less than pHPZC (pH_IEP_), Eq. 2 results in creation of the positive surface charge and positive zeta potential. When pH is larger than pH_PZC_ (pH_IEP_), Eq. 3 results in creation of the negative surface charge and negative zeta potential^22^,^23^,^24^. The dispersion hydrodynamic diameter is controlled by nanoparticle agglomeration in the aqueous system.

In the classical Derjaguin–Landau–Verwey– Overbeek (DLVO) theory, the agglomeration of nanoparticles is determined by the sum of the repulsive electrostatic force (the interaction of electrical double layer surrounding each nanoparticle) and the attractive van der Walls force^25^,^26^. Increase in particle surface charge (zeta potential) can enhance the electrostatic repulsive force, suppress the agglomeration, and subsequently reduce dispersion hydrodynamic size^27^. The effects of solution pH and ionic strength (IS) on nanoparticles dispersion properties are presented first, followed by discussion about the pH effect of particle size of ZnCr_2_O_4_ nanostructures.

**Figure 6:**
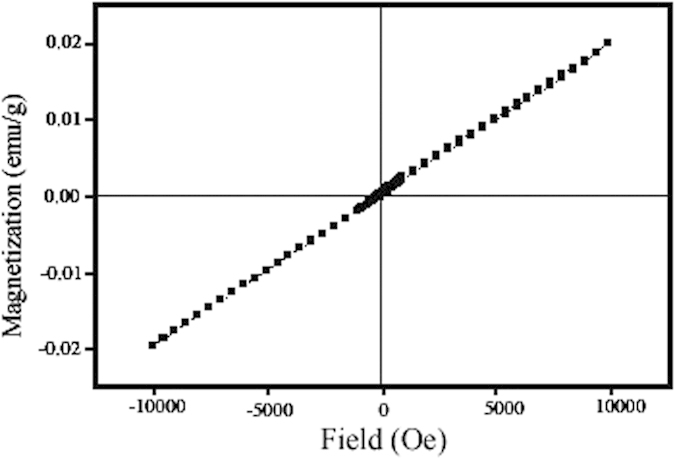
XRD patterns of sample (**a**) 8 and (**b**) 9.

Figure 6 is the XRD patterns of the sample 8 and 9 which obtained at pH 11 and 7. The product obtained at pH = 11 (sample 8) is cubic phase ZnCr_2_O_4_ (JCPDS card No. 73–1962), no significant diffractions of other phases can be found in the figure (Fig. 6a). A mixture of ZnCr_2_O_4_ (JCPDS card No. 73–1962) and ZnO (JCPDS card No. 80–0085) is produced with decreasing of pH to 7 (sample 9) shown in Fig. 6b. Hence, pH = 14 was chosen as an ideal pH for achieving pure and fine particles of ZnCr_2_O_4_.

**Figure 7:**
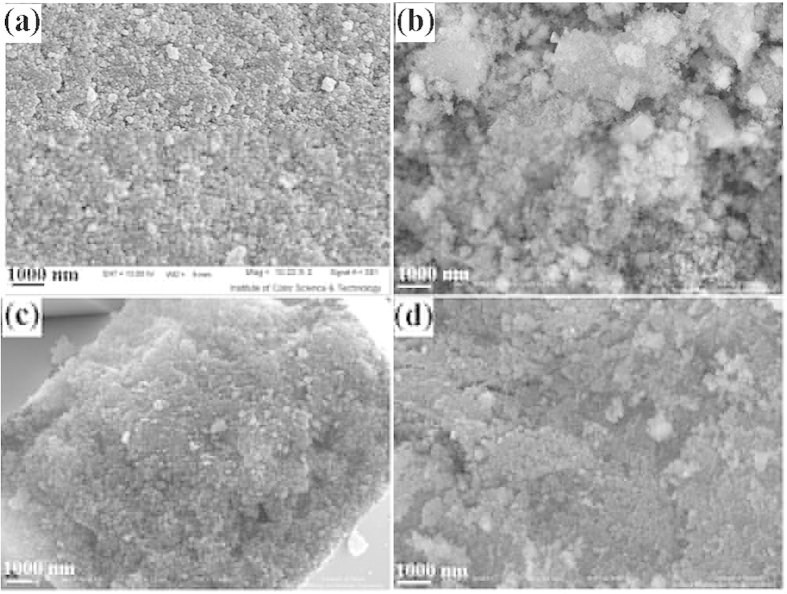
(**a**) FT-IR spectrum, (**b**) EDS pattern, and (**c**) PL spectrum of ZnCr_2_O_4_ (sample 4).

Figure 7a shows the FT-IR spectrum of ZnCr_2_O_4_ nanoparticles (sample 4). The two high frequency modes corresponding to peaks at 510 cm^−1^ and 625 cm^−1^ involve mainly the displacement of oxide anions relative to the chromium cations along the direction of the octahedral chains, and tentatively assigned to Cr (III) – O stretching^28^. The broad banding in the range 3000–3700 cm^−1^ belongs to the stretching vibrations of the coordinated water molecules.

Figure 7b shows the energy dispersive X-ray spectrum of ZnCr_2_O_4_ nanoparticles. This was carried out to investigate the chemical composition and the purity of ZnCr_2_O_4_ (sample 4). The strong peaks related to Zn, Cr and O are found in this spectrum. There was no unidentified peak observed in EDS. This confirms the purity and the composition of the ZnCr_2_O_4_ nanoparticles.

Figure 7c depicts the room-temperature photoluminescence emission spectrum of ZnCr_2_O_4_ nanoparticles, which was taken under excitation at 225 nm. The emission spectrum of sample 4 gives maxima at 312.5 nm. The determined band gap of this sample in ethanol solution is 3.96 eV.

**Figure 8:**
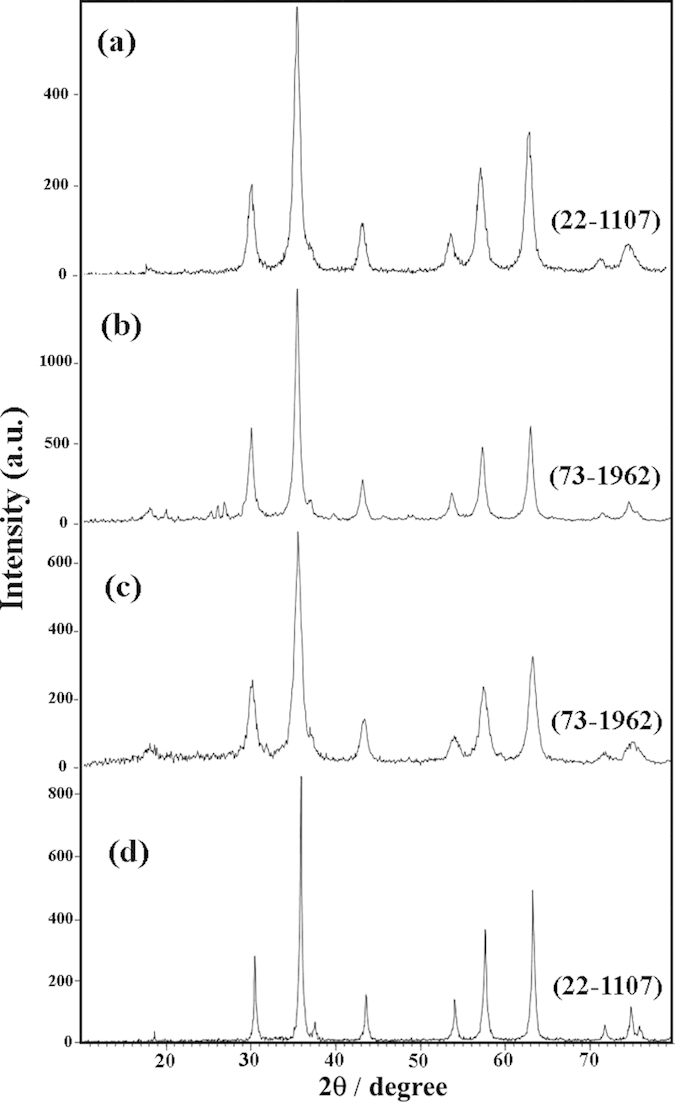
(Ahν)^2^ - hν curve for ZnCr_2_O_4_ nanoparticles (sample 4).

Figure 8 shows the (Ahν)^2^ - hν curve for ZnCr_2_O_4_ nanoparticles (sample 4). The band gap of the ZnCr_2_O_4_ particles is about 3.35 eV.

The band gap of ZnCr_2_O_4_ synthesized in this work was estimated about 3.35 eV and 3.96 eV by UV-vis and PL spectroscopy, respectively. As shown the amount of calculated band gap of the product using these methods (Tauc equation in UV-vis and 1240/λ_max_ in PL spectroscopy) is not same, this difference can be explained follow:

Usually, the traditional method of estimating of the band gap of semiconductor materials is based on the results of absorption spectra measurements. An evaluation of bandwidth is based only on the absorption measurements as a result of interband transitions. Fluorescence upconversion signal is the sum of two fluorescence beams, one from the sample itself (which is the actual fluorescence of molecule) and one from the excitation source. Up-converted signal is not due to the relaxation of molecular excited state or you cannot say that we have got a higher optical band gap. In case of two photon absorption, the absorption of the second photon comes from a virtual excited state, and not from actual excited state (LUMO or S1). So in both cases, the band gap of the molecule is same, as obtained by UV-visible spectroscopy. So, calculation of band gap through UV-vis spectroscopy is more accurate.

**Figure 9:**
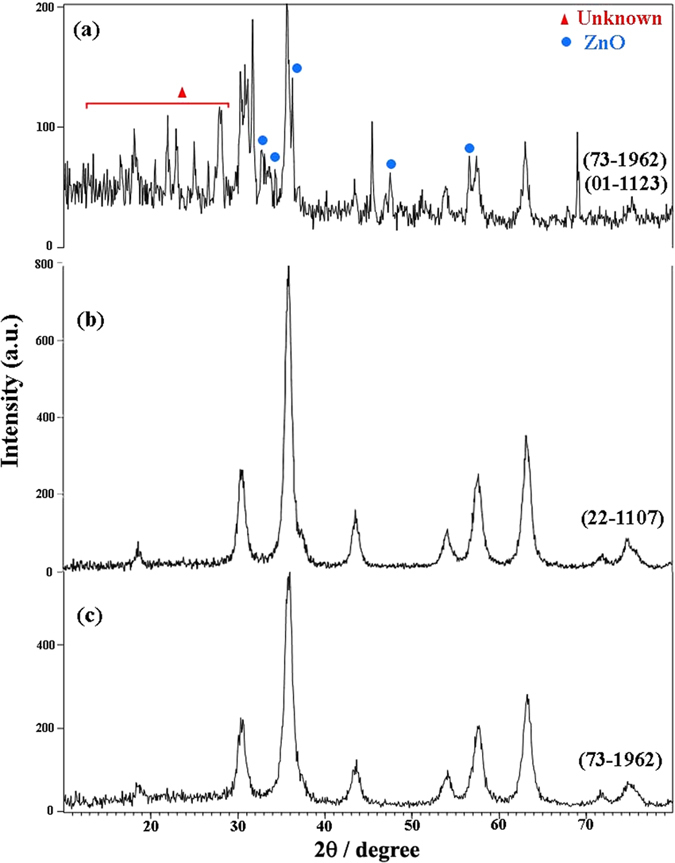
Photocatalytic activities of ZnCr_2_O_4_ nanoparticles (sample 4).

The photocatalytic activities of the obtained particles are evaluated by the decomposition of Eosin-Y and phenol red, as shown in Fig. 9. Under UV light irradiation, the ZnCr_2_O_4_ nanoparticles show apparent photocatalytic activities to the two organic pollutants, especially for Eosin-Y. The decomposition rate of Eosin-Y and phenol red reach 95.26% and 13.42 during UV-irradiation for 30 min, respectively. This reveals that ZnCr_2_O_4_ has the potential to be used as a new kind of semiconductor photocatalyst.

The photocatalytic activity mechanism of ZnCr_2_O_4_ was given in Eqs. 4, 5, 6, 7:


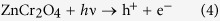



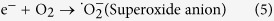



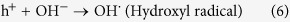



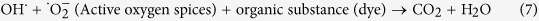


**Figure 10:**
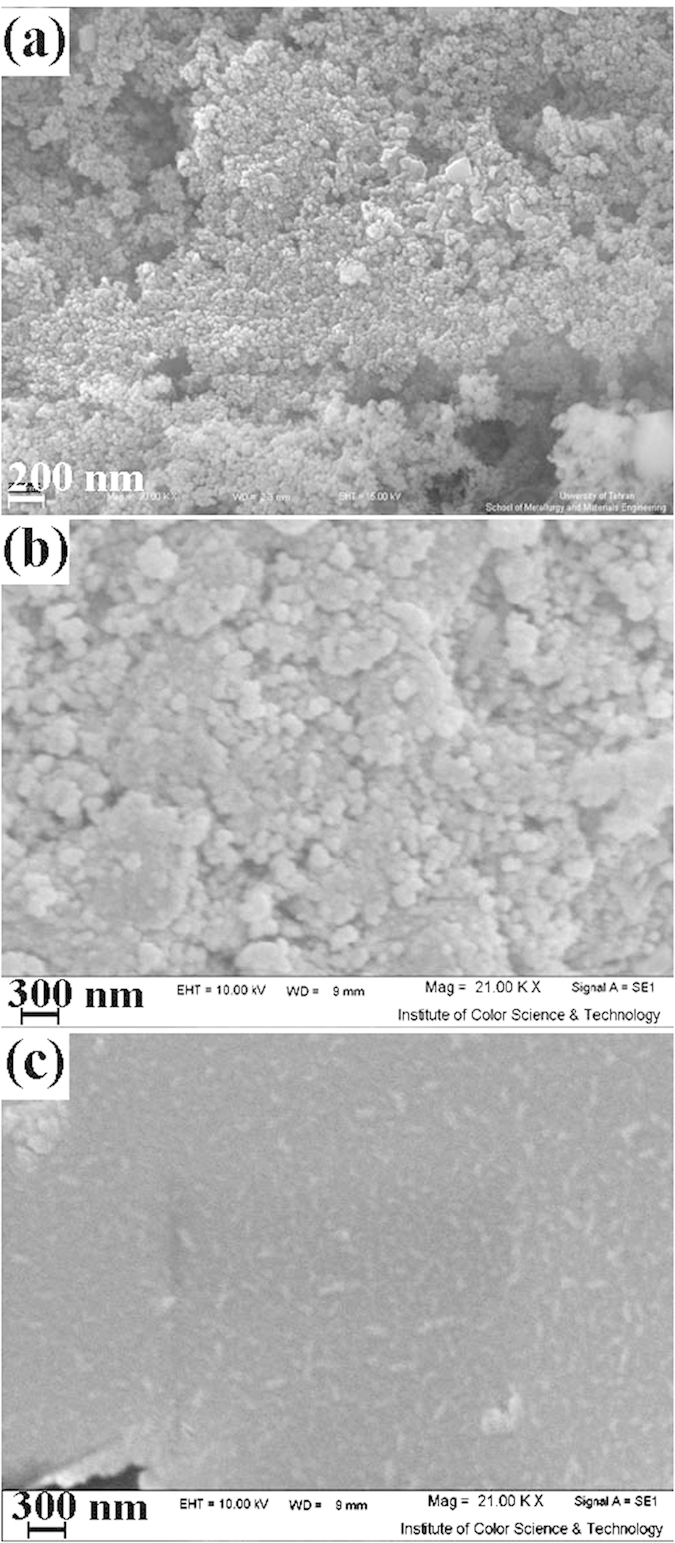
Photograph of measured contact angle (θ) on rough surface of zinc chromium oxide (pellet) material (sample 4).

Surface-wetting behaviors have recently attracted significant attention due to their potential photocatalytic applications. It is believed that surface energy, surface roughness, and surface chemical composition have strongly affected the surface wettability of the solid materials^29^, the obtained ZnCr_2_O_4_ nanoparticles show superhydrophilic behavior, which can be primarily attributed to the capillary effect^30^. Figure 10 shows the image of contact angle on the rough surface of zinc chromium oxide material. It was seen that a contact angle (θ) of zinc chromium oxide material is 5^ο^.

**Figure 11:**
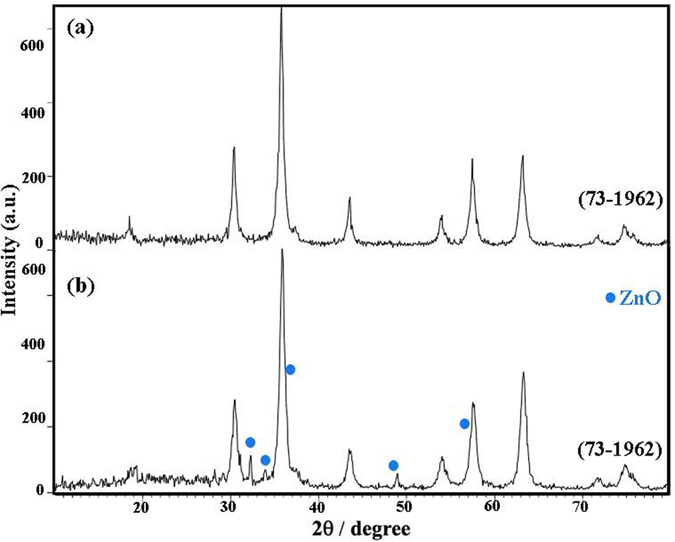
The M–H curve for sample 4 at room temperature.

The VSM measurement at room temperature was carried out to understand the magnetic characteristics of ZnCr_2_O_4_. Figure 11 shows the M–H curve for ZnCr_2_O_4_ nanoparticles (sample 4). It is evidenced from the hysterical behavior of the M–H curve that all the nanoparticles are paramagnetic at room temperature, although bulk ZnCr_2_O_4_ is an antiferromagnetic compound^31^. Changing magnetic property of ZnCr_2_O_4_ in two scales, bulk and nano can be ascribed to finite size effects^32^. It was shown that the magnetic moment per atom and the magnetic anisotropy of nanoparticles can be different than those of a bulk specimen.

The two main features that dominate the magnetic properties of nanoparticles and give them various special properties are:

(a) Finite-size effects (single-domain or multi-domain structures and quantum confinement of the electrons);

(b) Surface effects, which results from the symmetry breaking of the crystal structure at the surface of the particle, oxidation, presence of defects, dangling bonds, fluctuations in the number of atomic neighbors and lattice expansion cause disorder of surface spin and frustration^33^.

When the magnetic materials obey quantum confinement effect that their dimensions become less than the size of magnetic domain. In large magnetic particles, it is well known that there is a multi-domain structure where regions of uniform magnetization are separated by domain walls. If the particle size is reduced, there is a critical volume below which it costs more energy to create a domain wall than to support the external magnetostatic energy (stray field). Under this critical diameter, which typically lies in the range of a few tens of nanometers (and depends on the type of material), the particle will consist of a single domain, single domain particles are the ferromagnetic material cannot split up further into domains^34^,^35^,^36^.

The MNP might be composed of a single magnetic domain and it might also display a superparamagnetic^37^,^38^ behavior. In the superparamagnetic state, the magnetic moments of the nanoparticles fluctuate around the easy axes of magnetization. Thus, each one of the MNPs will possess a large magnetic moment that continuously changes orientation. When a magnetic field is applied, MNPs in the superparamagnetic state display a fast response to the changes in the magnetic field without remnant (residual) magnetization and without coercivity (the magnetic field required to bring the magnetization back to zero). Thus, in the superparamagnetic state, the MNP behaves as a paramagnetic atom with a giant spin^39^.

## Conclusions

In summary, ZnCr_2_O_4_ nanoparticles have been successfully synthesized from Zn(NO_3_)_2_. 6H_2_O and CrCl_3_. 6H_2_O by a simple co-precipitation method, under low temperature and ambient pressure. The fine and pure ZnCr_2_O_4_ nanoparticles were produced through adjusting the pH value, appropriate alkaline and capping agents. Wettability of ZnCr_2_O_4_ nanoparticles synthesized in this work, was considered by the contact angle goniometer. The contact angle (θ) was 5^ο^, which indicates that oxide material was superhydrophilic in nature. In comparison to other similar works, our method is facile, simple, low cost, and eco-friendly. The produced zinc chromite nanostructures can be utilized as a remarkable photocatalyst for dye degradation from waste-water such as removal of Eosin-Y and phenol red as water pollutants under UV irradiation. The degradation percentages of Eosin-Y and phenol red under UV-irradiation were calculated about 95.26 and 13.42, respectively. VSM analysis depicted that ZnCr_2_O_4_ nanoparticles have a paramagnetic characteristic, although the bulk ZnCr_2_O_4_ represents an antiferromagnetic behavior and this change in magnetic property can be ascribed to finite size effects in nano scale materials.

## Methods

### Synthetic procedure

ZnCr_2_O_4_ nanostructures were prepared by reacting between Zn(NO_3_)_2_. 6H_2_O and CrCl_3_. 6H_2_O as starting materials with molar ratio is equal to 1: 2, respectively. At first, 0.20 g of Zn(NO_3_)_2_. 6H_2_O as zinc source and 0.2 g of capping agent were dissolved in distilled water and then, a solution including 0.35 g of CrCl_3_. 6H_2_O was added to above solution under vigorous magnetic stirring for 20 min. The alkaline solution in order to reach proper pH was slowly added to the former solution, then the obtained mixture was heated at 60 °C for 30 min. The products were washed and dried under vacuum at 70 °C. Finally, the as-prepared precipitates were calcined in air at 700 °C for 3 h.

## Experimental details

### The preparation mechanism of ZnCr_2_O_4_ nanoparticles

The proposed mechanism for the synthesis of ZnCr_2_O_4_ nanoparticles could be explained as follows by Eqs. 8, 9, 10:


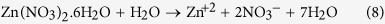



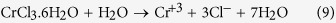



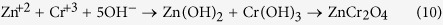


### Determination procedure of optical band gap

The optical band gap (E_g_) of semiconductors can be calculated on the basis of the optical absorption spectrum by the equation 11:


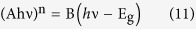


where hν is the photo-energy, A is absorbance, and B is a constant relative to the material when n depends on whether the transition is direct (n = 2) or indirect (n = 1/2)^40^,^41^. The optical band gap for the absorption peak can thus be deducted by extrapolating the linear portion of the (Ahν)n - hν curve to zero. From the function curve of (Ahν)^1/2^ - hν, no linear relation was found, indicating that the as-prepared ZnCr_2_O_4_ sample is a direct band gap semiconductor.

### Procedure of photocatalytic experiment

In a typical experiment, 0.1 g of dye solution and 0.05 g of ZnCr_2_O_4_ as a photocatalyst were mixed using a magnetic stirrer. After a period of time in the dark, the solution was irradiated. The light source was the Osram ultraviolet lamp with a power of 400 W that placed at a fixed distance of 40 cm away from the reaction vessel. The radiation source of ultraviolet (UV) is very important for photocatalytic activities. Ultraviolet radiation refers to electromagnetic radiation in the 200–400 nm wavelength range. UV-A covers from 315 to 400 nm, UV-B from 280 to 315 nm and UV-C from 200 to 280 nm. Artificial UV lamps can power photocatalytic processes. The band gap of ZnCr_2_O_4_ is about 3.5 eV, so UV source should be provided light irradiation in this range, therefore UV-A (λ_max_ is about 365 nm) is appropriate for the photocatalytic activity of this product. To maintain the solution oxygen-saturated throughout the reaction, air was blown into the vessel via a pump. The light source and quartz vessel were placed in a black box that equipped with a fan. Aliquots of the mixture were taken at definite interval of times during the irradiation, and after centrifugation; they were analyzed by a UV–vis spectrometer. The dyes degradation percentage was calculated as follows in equation 12:


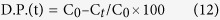


where C_0_ and C_t_ are the absorbance value of the solution at 0 and t min, respectively.

## Characterization

Cetyltrimethyl ammonium bromide (CTAB), sodium dodecyl-benzene-sulfonate (SDBS), and poly vinyl pyrrolidone (PVP-25000) were purchased from Merck. The XRD of products was recorded by a Rigaku D-max C III XRD using Ni-filtered Cu Kα radiation. SEM images were obtained on Philips XL-30ESEM equipped with an energy dispersive X-ray spectroscopy. The EDX analysis with 20 kV accelerated voltage was done. Room temperature PL was studied on a Perkin Elmer (LS 55) fluorescence spectrophotometer. Fourier transform infrared (FT-IR) spectrum was recorded on Shimadzu Varian 4300 spectrophotometer in KBr pellets. The magnetic properties of the samples were detected at room temperature using a vibrating sample magnetometer (VSM, Meghnatis Kavir Kashan Co., Kashan, Iran).

## Additional Information

**How to cite this article**: Mousavi, Z. *et al.* ZnCr_2_O_4_ Nanoparticles: Facile Synthesis, Characterization, and Photocatalytic Properties. *Sci. Rep.* **6**, 20071; doi: 10.1038/srep20071 (2016).

## Figures and Tables

**Table 1:** The reaction conditions for synthesis of ZnCr_2_O_4_ via a simple co-precipitation. method.

Sample	Capping agent	pH	Alkaline agent
1	(blank)	14	NaOH
2	PVP-25000	14	NaOH
3	CTAB	14	NaOH
4	SDBS	14	NaOH
5	SDBS	14	NH_3_
6	SDBS	14	Ethylene diamine (en)
7	SDBS	14	Propylene diamine (pn)
8	SDBS	11	NaOH
9	SDBS	7	NaOH
