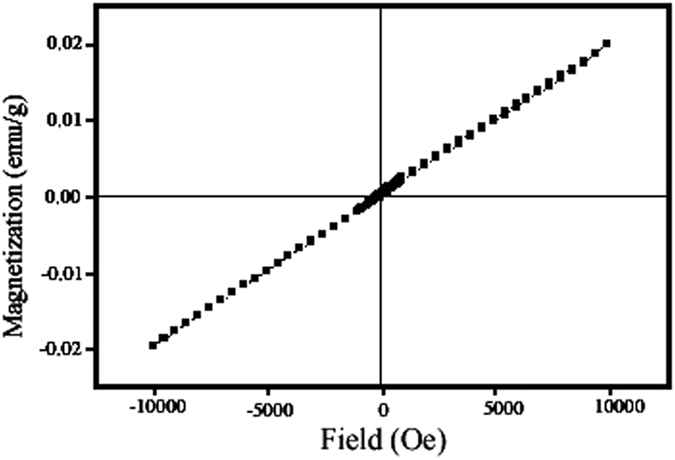# Corrigendum: ZnCr_2_O_4_ Nanoparticles: Facile Synthesis, Characterization, and Photocatalytic Properties

**DOI:** 10.1038/srep25862

**Published:** 2016-05-23

**Authors:** Zahra Mousavi, Faezeh Soofivand, Mahdiyeh Esmaeili-Zare, Masoud Salavati-Niasari, Samira Bagheri

Scientific Reports 6: Article number: 2007110.1038/srep20071; published online: 02012016; updated: 05232016

This Article contains an error in the order of the Figures. Figure 1, 2, 3, 4, 5, 6, 7, 8, 9, 10 and 11 were published as Figure 7, 8, 1, 9, 10, 11, 2, 3, 4, 5 and 6 respectively. The correct Figures appear below as Figures 1, 2, 3, 4, 5, 6, 7, 8, 9, 10 and 11. The Figure legends are correct.

## Figures and Tables

**Figure 1:**
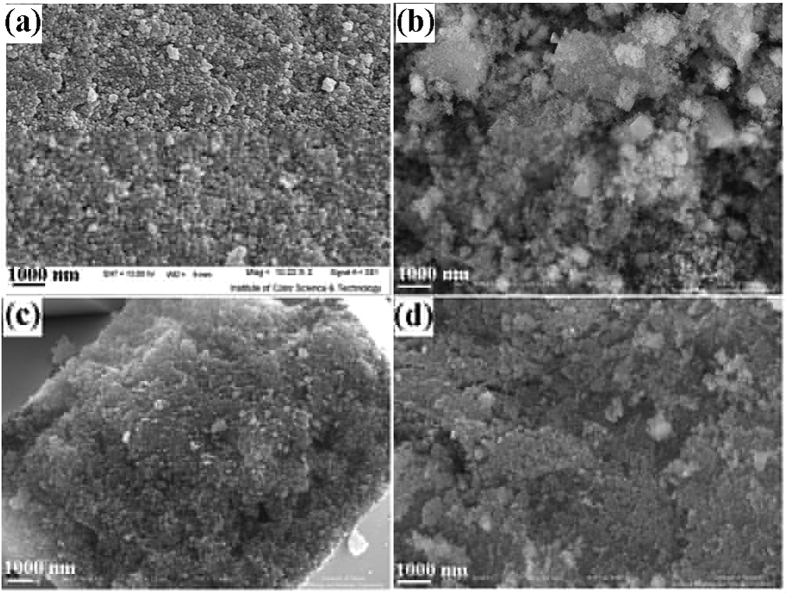


**Figure 2:**
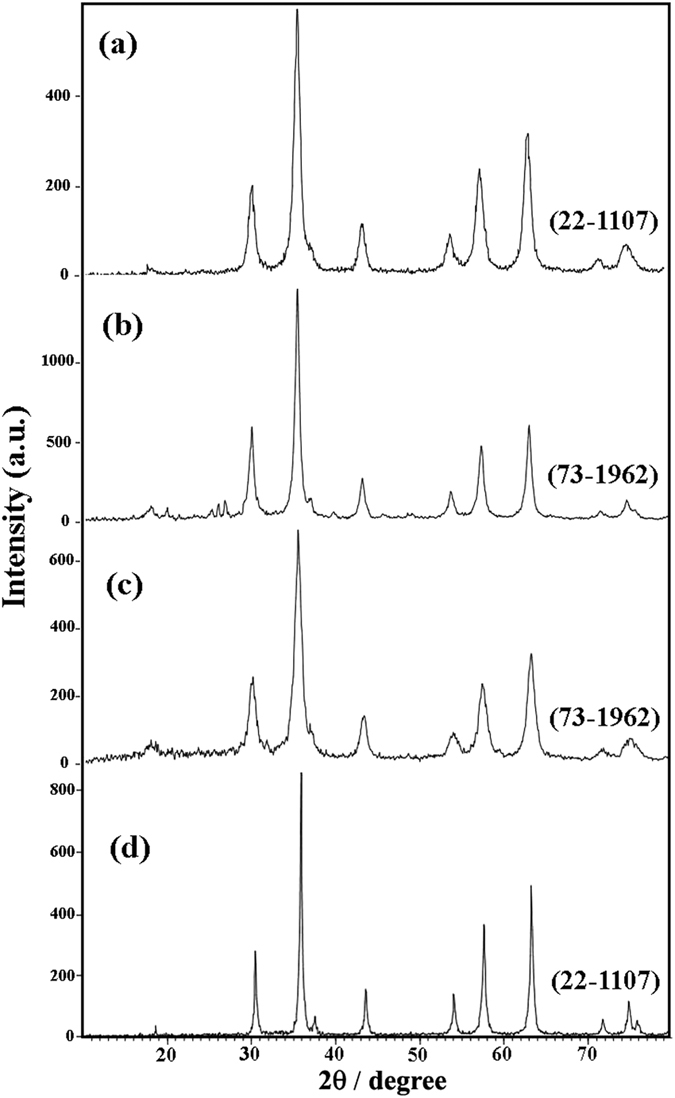


**Figure 3:**
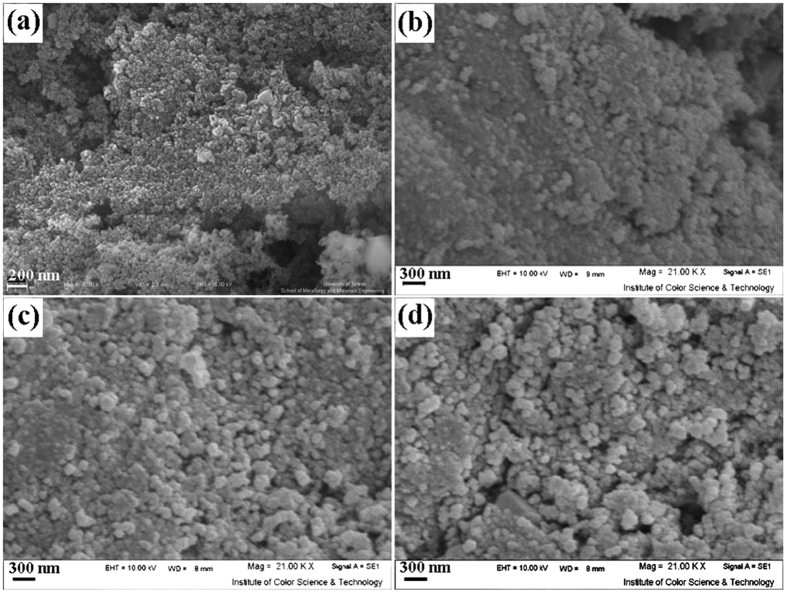


**Figure 4:**
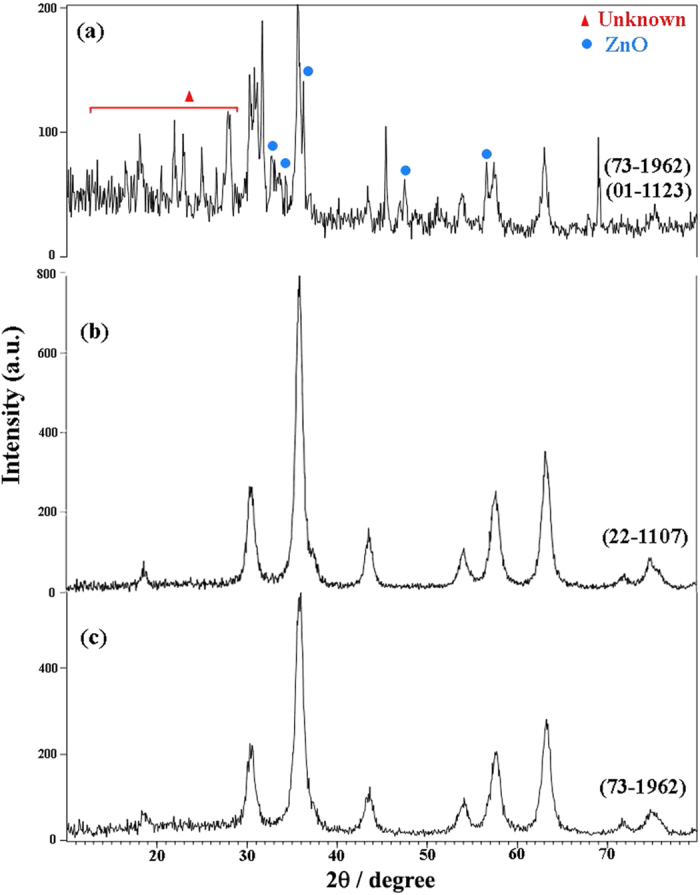


**Figure 5:**
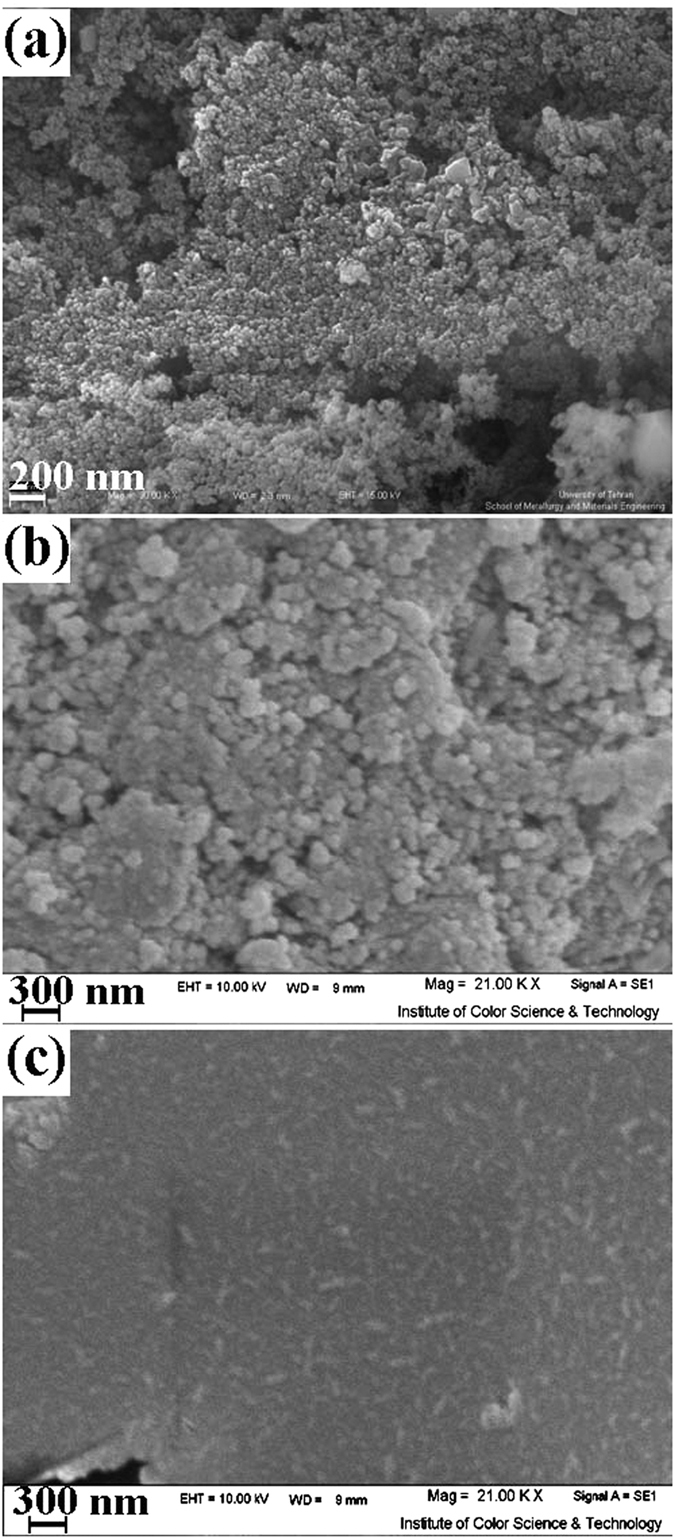


**Figure 6:**
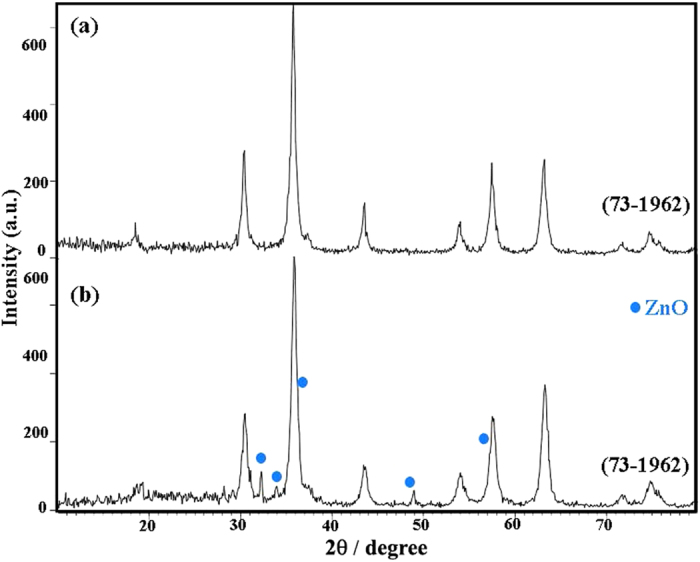


**Figure 7:**
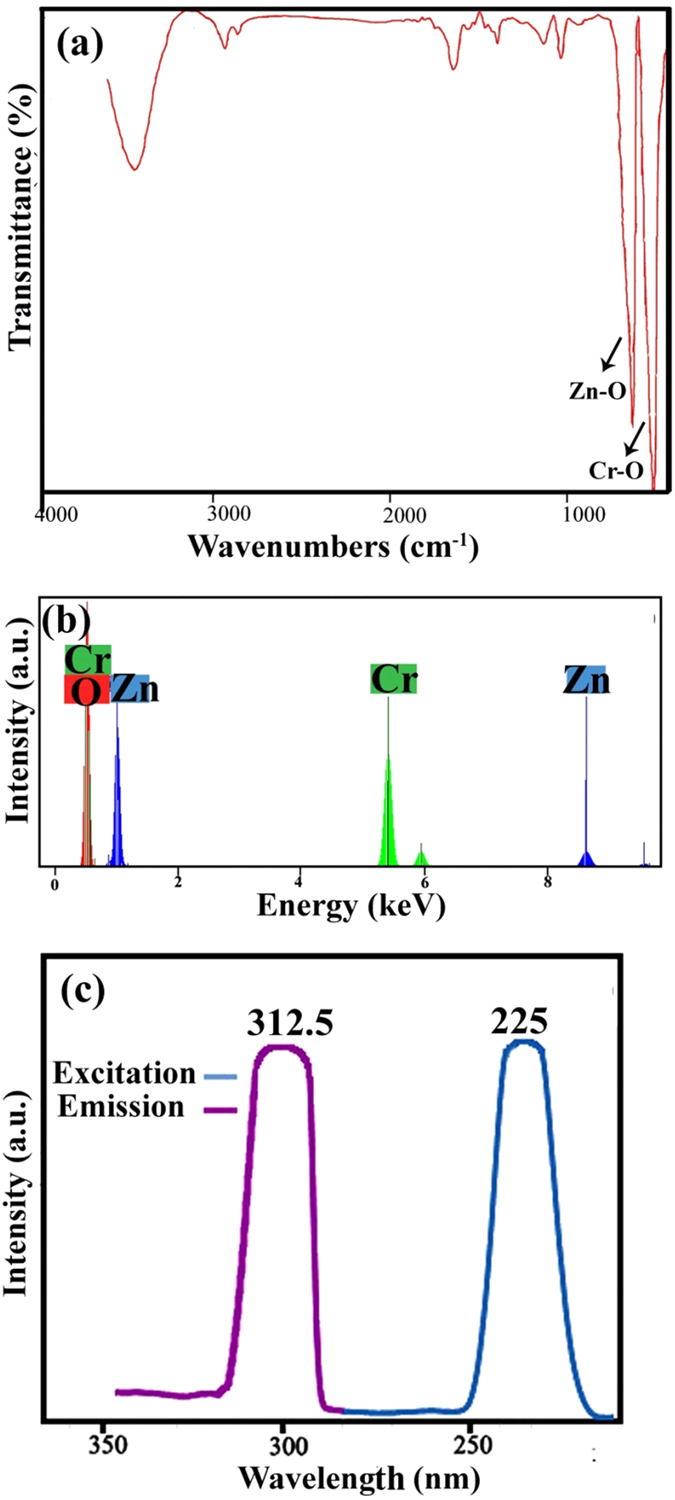


**Figure 8:**
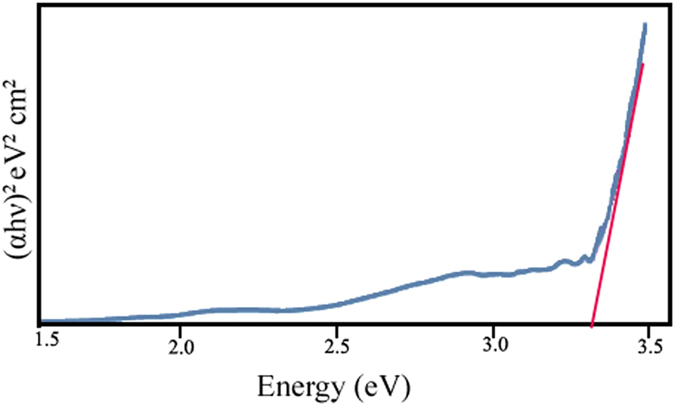


**Figure 9:**
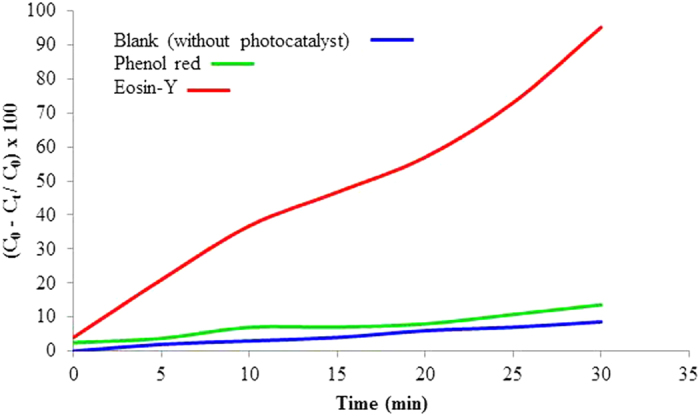


**Figure 10:**
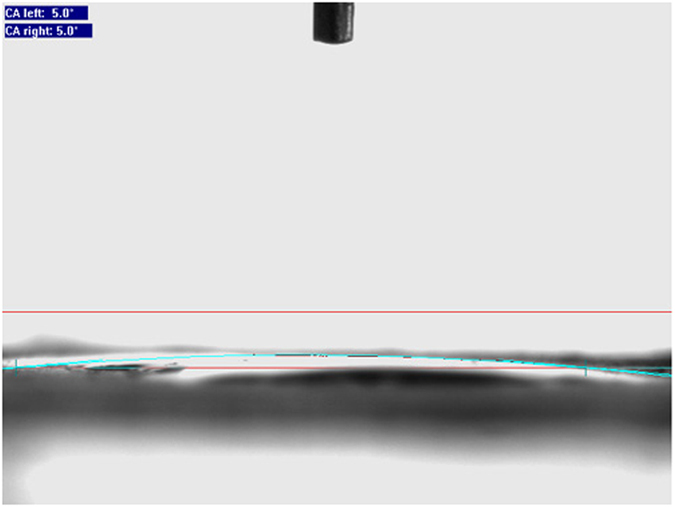


**Figure 11:**